# Jumping to Conclusions Is Associated with Paranoia but Not General Suspiciousness: A Comparison of Two Versions of the Probabilistic Reasoning Paradigm

**DOI:** 10.1155/2012/384039

**Published:** 2012-10-18

**Authors:** Steffen Moritz, Niels Van Quaquebeke, Tania M. Lincoln

**Affiliations:** ^1^Department of Psychiatry and Psychotherapy, University Medical Center Hamburg-Eppendorf, Martinistraße 52, 20246 Hamburg, Germany; ^2^Department of Management and Economics, Kuehne Logistics University, Hamburg, Germany; ^3^Department of Psychology, University of Hamburg, Hamburg, Germany

## Abstract

Theoretical models ascribe jumping to conclusions (JTCs) a prominent role in the pathogenesis of paranoia. While many earlier studies corroborated this account, some newer investigations have found no or only small associations of the JTC bias with paranoid symptoms. The present study examined whether these inconsistencies in part reflect methodological differences across studies. The study was built upon the psychometric high-risk paradigm. A total of 1899 subjects from the general population took part in an online survey and were administered the Paranoia Checklist as well as one of two different variants of the probabilistic reasoning task: one variant with a traditional instruction (a) and one novel variant that combines probability estimates with decision judgments (b). Factor analysis of the Paranoia Checklist yielded an unspecific suspiciousness factor and a psychotic paranoia factor. The latter was significantly associated with scores indicating hasty decision making. Subjects scoring two standard deviations above the mean of the Paranoia Checklist showed an abnormal data-gathering style relative to subjects with normal scores. Findings suggest that the so-called decision threshold parameter is more sensitive than the conventional JTC index. For future research the specific contents of paranoid beliefs deserve more consideration in the investigation of decision making in schizophrenia as JTC seems to be associated with core psychosis-prone features of paranoia only.

## 1. Introduction

Research on neuropsychological dysfunctions in schizophrenia (e.g., memory and executive dysfunction) has been increasingly extended by studies on cognitive biases [1–3]. Cognitive biases represent preferences, subtle distortions, and styles of information processing rather than neural deficits or mere inaccuracy [1]. An emerging literature has elucidated that persons with delusions tend to jump to conclusions [3, 4], are over-confident in their incorrect decisions [5–9], and show attributional biases [10–12], for example, a preference for monocausal inferences [13], and a bias against disconfirmatory evidence [14–18]. Some of these biases have been found to correlate with positive symptoms (i.e., delusions and hallucinations), which according to many clinicians represent the core of the disorder. Cognitive training programs such as the Social Cognition and Interaction Training (SCIT) [19, 20], the Maudsley Review Training Program [21] or the Metacognitive Training for Psychosis (MCT) [22, 23] have begun to translate these insights into practice: patients learn to withhold strong judgment in the face of ambiguous evidence and to be more flexible in their decision making.

The present study is primarily concerned with JTC which is usually assessed with the so-called beads or probabilistic reasoning task [24]. This task requires the subject to deduce from which of two containers a string of beads has been drawn. Typically, containers contain beads in an opposing ratio (e.g., 90 : 10 versus 10 : 90). Decisions after only one or two beads are counted as evidence for JTC. Since its introduction, numerous variants have been developed using different numbers of containers, material (e.g., beads/containers, fish/lakes, adjectives/personality traits, sheep/herds), mode of administration (e.g., real beads/containers versus computerized tasks), ratios (e.g., 90 : 10, 85 : 15, 80 : 20) and types of response (simulated decisions, probability assessment or concurrent measurement of probabilities and decisions) or manipulated other facets of the task. Notwithstanding these alterations, the evidence for JTC has been quite robust across many studies: numerous investigations found an association of JTC with (paranoid) delusions or dimensions of delusions, such as delusion conviction [25–30]. While an older review of Fine et al. [4] concludes that “a tendency to gather less evidence in the beads task is reliably associated with the presence of delusional symptomatology” (p. 46), some (more recent) studies have not been able to detect substantial associations with paranoid or delusional symptomatology [31–34] and there is mixed evidence whether nonclinical subjects scoring high on paranoia display JTC [35, 36]. In one study, JTC was pronounced in currently deluded patients but still detectable in nondeluded ones [37]. In contrast, Lincoln and colleagues [29] found JTC in acute but not remitted patients, whereby the association between JTC and delusions vanished when negative symptoms were accounted for. Few studies have looked at the impact of different themes of delusions on jumping to conclusions. An earlier study from the group of Garety and Freeman reported a rather specific association between JTC and persecutory delusions [38], whereas a novel study of the same group [39] found that grandiose delusions were more associated with JTC than persecutory delusions (as assessed with a 85 : 15% variant).

Two large recent studies are particularly noteworthy in our view as they were unable to secure substantial differences on the prevalence of JTC in patients versus controls. One study [40, 41] on 85 patients with schizophrenia and 25 healthy controls revealed no greater JTC in patients than in controls. Moreover, the amount of JTC was not moderated by the severity of positive symptomatology. Likewise, in the to date largest study on cognitive biases in schizophrenia (*N* = 289 patients), patients showed more JTC relative to 55 controls only at statistical trend level [42]. Correlations with positive symptoms were significant but small. 

The two aforementioned studies both used a more complex version of the probabilistic reasoning task so that methodological differences to the conventional task may at least partially account for the inconsistent findings. First, the task used fish/lakes instead of beads/jars [30] which, however, can be regarded as a minor modification as this setup is quite intuitive and has elucidated group differences in other studies [43]. Perhaps more important, the novel task requested subjects to perform two judgments: first they were asked to provide an estimate of the probability that a (sequence of) fish is from lake A or B and then whether they would decide for one of the lakes or not. The parallel assessment of probability and decision serves the purpose to assess the decision threshold of schizophrenia patients as it has been put forward that people with schizophrenia may reason like *bad statisticians *who do not rely on high levels of probability before a decision is accepted (e.g., 95% as usually in statistics), but are satisfied with lower levels [6, 44–46]. A lowered acceptance threshold automatically increases the number of erroneous hypotheses. In line with this contention, different studies have demonstrated that patients with schizophrenia had a much lower significance threshold (i.e., liberal acceptance account). For example, in a study modeled after the “Who wants to be a millionaire” quiz [47], patients had a minimal decision threshold of 54% versus 70% in controls (mean decision threshold: 86% versus 93%) which resulted in a higher error rate. A low decision threshold has been replicated using other tasks as well and has also been found to be rather specific to schizophrenia patients [48]. Interestingly, a low decision threshold has been shown to be more sensitive to group differences than the original JTC parameters, such as draws to decision [49]. While we deem the measurement of decision thresholds an important advancement in the research on reasoning in schizophrenia, asking subjects to estimate probabilities might have fostered additional checks of the available evidence and thereby delayed decisions. Moreover, unlike the traditional variant this task did not request high confidence in the decision in order to dissociate probability and decision making. It may be argued that overconfidence in a quick decision may be more pathological and potentially discriminating across groups than just any quick decision [50]. 

Another reason for the heterogeneity of findings may relate to the different methods used to measure paranoia. While some studies used expert ratings like the Positive and Negative Syndrome Scale (PANSS); [51], others have used self-rating scales like the Peters' Delusions Inventory (PDI); [52] or the Paranoia Checklist [53]. Perhaps more importantly, paranoia is a multidimensional phenomenon that can be characterized along different aspects like content (e.g., suspiciousness versus Schneiderian symptoms), conviction, distress, and impact on behavior [54]. It deserves consideration that the JTC bias might be associated with some but not all of these aspects. 

The present study was the first to conduct a head-to-head comparison of a version modeled after the traditional variant of the beads task [24] and a new paradigm that assesses probability estimates and decision judgments within one task [43]. We were interested to see if the pattern of results and psychopathological correlates is comparable across tasks. 

Based on the notion that paranoia is represented along a continuum in the general population and some evidence [25, 36, 55] for a “dose-response relationship” between level of psychosis liability and JTC [35], we adopted a so-called psychometric high risk approach: subjects selected from a large population sample scoring higher than two standard deviations (SDs) above the mean of the Paranoia Checklist (see Section 2) were compared to a sample with scores not higher than 0.5 standard deviations above the mean [25, 56, 57]. The second aim was to examine if JTC is related to specific aspects of paranoia. To meet this purpose, we conducted a factor analysis on the Paranoia Checklist and correlated core parameters of the probabilistic reasoning tasks with the factor scores.

## 2. Methods

### 2.1. Participants and Procedure

Participants were recruited via the WiSo-panel, an academic online service in Germany that allows researchers to advertise their studies to potential participants. Recent research has demonstrated that this and similar services (e.g., Mturk or Studyresponse in the USA) provide reliable means of collecting data [58–60].A total of 7,947 subjects from the general population were invited for participation in the study which was conducted over the internet using the software package “Unipark” [61]. Of these, 1,899 (24%) completed all tasks relevant for the present study. Subjects' age, gender, and educational level were drawn from the WiSo-panel data base. Before the experiments started, subjects were asked to provide informed consent. Then, the survey proceeded with items on perceptions of ethical leadership in organizations, all of which are unrelated to the present study and will be presented elsewhere. After that, the 18-item Paranoia Checklist [53] was administered which assesses paranoid beliefs. Good psychometric properties of the German version have been demonstrated in previous studies [62, 63]. At the end of the survey, each subject was randomly presented with one out of two versions of the fish task. For both versions the order of events was fixed mirroring the primary ratio of the lakes (both 80 : 20% versus 20 : 80%; the fourth and the ninth fish were in the color of the nondominant lake (lake B)). Upon a decision for lake A or B (the subject could decide either for lake A or B or make no decision) the task and the entire study were terminated. In both variants each new fish was highlighted with an arrow and shown along with previously caught fish. Moreover, the ratio of fish was explicitly shown on each slide to minimize the influence of memory. The two versions were as follows.

**(a) For the Traditional Variant, the Subject Was Shown the Following Instruction**

“Below you see two lakes with red and green fish. Lake A: 80% red fish and 20% green. Lake B: 80% green fish and 20% red. A fisherman randomly chooses one of the two lakes and then fishes from this lake only. Based on the caught fish, you should decide whether the fisherman caught fish from lake A or B. Important: (1) The fisherman catches fish from one lake only. (2) He throws the fish back after each catch. The ratio of green and red fish stays the same. (3) You can catch as many fish as you need to be completely sure as to which lake the fisherman has chosen.”

**(b) In the Extended Condition, Subjects Were Provided the Following Instruction (High Confidence in the Decision Was Not Explicitly Requested)**

“Below you see two lakes with orange and gray fish. Lake A: 80% orange fish and 20% gray. Lake B: 80% gray fish and 20% orange. A fisherman randomly chooses one of the two lakes and then fishes from this lake only. After each new catch, please make the following judgments: (1) What is the probability that the fish are being caught from lake A or lake B (0–100%)? (2) Do you have enough information to decide on one particular lake? Important: The fisherman catches fish from one lake only. He throws the fish back after each catch. The ratio of orange and gray fish stays the same.”

As outlined before, we adopted a psychometric high-risk approach [56], whereby the performance of participants scoring at least 2 standard deviations above the mean of the Paranoia Checklist is contrasted with participants with scores not higher than 0,5 SD above the mean of the sample.

## 3. Results

### 3.1. Background and Experimental Data

Demographic, psychopathological, and experimental characteristics of the sample are displayed in Table 1. Most participants were female, around 40 years old and had a high educational level. The Paranoia Checklist total score was comparable to prior studies [64, 65]. No differences emerged between subsamples (i.e., the subsamples undergoing the traditional versus the extended version) on background and psychopathological scores. However, there were significant differences between the traditional and the extended version of the fish task: the rate of JTC was higher in the extended variant relative to the traditional task. Accordingly, the number of draws-to-decision (DTD) was lower in the extended task. The minimal probability required for a decision in the extended task was 74%. This parameter could be computed for the extended variant only. 

Table 2 contrasts performance on the probabilistic reasoning tasks for participants who scored at least two standard deviations above the mean of the Paranoia Checklist and those with scores below a cut-off point of half a standard deviation above the mean. All comparisons were significant indicating a higher JTC bias, a smaller number of DTD, and a lowered decision threshold in the high scorers. In the novel variant, participants with high paranoia scores provided lower probability ratings after fish #1. 

### 3.2. Correlations

To explore whether core parameters of the probabilistic reasoning task are differentially correlated with paranoia, we submitted the Paranoia Checklist to a principal component analysis with varimax rotation. Both the scree-plot and the Kaiser-Guttmann criterion (extraction of factors with eigenvalues > 1) suggested a two-factor solution (see Table 3) which explained approximately two thirds of the variance (64%). The Bartlett's test of sphericity was significant, *χ* ^2^(153) = 25566,82; *P* < .001. The Kaiser-Meyer-Olkin measure suggested a good fit (.95). Factor 1 (eigenvalue = 6.63) explained 37% of the variance and was named “unspecific suspiciousness” as it was primarily loaded by (low threshold) items covering “normal” suspicion such as “There might be negative comments being circulated about me.” The second factor (eigenvalue = 4.90) explained 27% of the variance and was named “psychotic paranoia” as it was primarily loaded by (high-threshold) items covering clearly pathological forms of delusions such as “I can detect coded messages about me in the press/TV/radio.” These items dealt with conspiracy and Schneiderian first-rank symptoms (i.e., permeability of ego boundaries). Factor scores of the Paranoia Checklist were saved to the matrix and correlated with the JTC measures.  Table 4 shows that none of the probability reasoning parameters correlated substantially with the first factor. In fact, the correlations for the traditional variant were small (*rs* < .1) but negative, that is, in the opposite direction than expected (i.e., higher questionnaire scores were related to lower JTC). Vice versa, the second factor was correlated with all JTC indexes in the predicted direction. The decision threshold was the only parameter that was also correlated with the Paranoia Checklist total score. All correlational differences for the two factor scores with the experimental parameters were (highly) significant (at least *P* < .05). 

## 4. Discussion

The present work was motivated by recent studies [41, 42] suggesting that JTC in schizophrenia may not be as robust as long claimed. We set out to investigate whether this line of research reflects an instance of the so-called decline effect [66] or methodological differences across studies. As mentioned before, the psychopathological correlates of JTC are not fully uncovered. We were especially interested in the question of whether the traditional instruction of the probabilistic reasoning task [24] would be more potent than a new variant of the task which requires the joint assessment of probability judgments and decisions. Recently, this extended variant produced some conflicting findings which—among other yet unknown factors—may mirror two things: first, asking for subjective probability may caution subjects and thus delay decision making even in otherwise hasty individuals. Secondly, the extended variant does not request high confidence for judgments so that even more cautious individuals may well decide after only few items. 

Results show that the prevalence of JTC was higher in the extended condition challenging the hypothesis that asking for probability levels cautions subjects and attenuates the level of JTC. Overall, the correlational pattern of experimental variables with paranoid delusions or the difference between high and normal scorers was comparable across tasks. Interestingly, even the traditional version produced a higher prevalence of JTC than is usually expected, which might be due to the administration mode over the internet. 

Factor analysis of the Paranoia Scale yielded two factors that resemble the two subscales of the Green et al. Paranoid Thought Scales (GPTS) [67]: a core paranoid dimension and a dimension measuring suspiciousness/social reference. The most important result in our view was that the JTC bias is apparently not correlated with normal suspiciousness (e.g., “There might be negative comments being circulated about me”) but rather with more psychopathological and psychosis-prone forms of paranoia (e.g., “My actions and thoughts might be controlled by others”). High scorers on the former dimension did not differ significantly from subjects in the normal range, whereas high scorers on the latter scale had a much greater JTC bias and lower decision threshold than those in the normal range. This finding corroborates recent research [68] that JTC is tied to psychotic themes: patients with obsessive compulsive disorders low on illness insight did not display a JTC bias.

A number of limitations need to be acknowledged. First, the study was conducted over the internet and none of the assessments was determined face-to-face. The sample consisted predominantly of females with higher education which markedly deviates from a clinical schizophrenia population (mainly male with low school achievements). While online research contains many advantages relating to economy and anonymity (e.g., subjects are perhaps more open to disclose psychological problems), results need verification in a conventional setting. However, prior results [69] and the fact that the Paranoia Checklist scores were in accord with mean values previously collected for nonclinical subjects [64, 65] tentatively speak for the validity of our findings. A more general problem with the probabilistic reasoning task is that it estimates the core parameter, JTC, by only a single item. The results of such “single shot” experiments are plagued by low reliability. This may be one reason why some studies using the traditional variant did not detect strong JTC in paranoid schizophrenia across all variants of the probabilistic reasoning task [68]. Alternative measures [25, 43, 70, 71] may be better in this regard and should at least complement the administration of the probabilistic reasoning task. One may also investigate whether another version of the beads task which separates ratings for the two choices [72] is more sensitive to paranoia. Finally, our study cannot fully answer the question why two studies using the extended variant failed to find strong support for JTC in schizophrenia. We can only speculate that perhaps levels of paranoia/delusions were lower in patients relative to the high-risk sample of the present study: paranoid symptoms wax and wane and patients with schizophrenia do not necessarily show high levels of positive symptoms throughout. 

For future studies we recommend to test additional potential moderators. According to some studies patients with schizophrenia and even some controls have problems to grasp the task instruction [37] and several studies have found that a JTC bias is associated with lower intelligence [29]. It also remains unresolved whether JTC is associated with delusions in general or only special subtypes (see Introduction). 

## 5. Conclusions

While our study showed that both versions of the probabilistic reasoning task are equally sensitive for JTC measurement per se, we argue that the extended version has an additional advantage: with the decision threshold it provides an important novel index that can pinpoint whether absence of differences on DTD or JTC may derive from differences on overall probability levels. As Table 2 shows, high-paranoid subjects display lower initial probability levels than those scoring in the normal range. Baseline differences can have a huge impact on the conventional JTC parameter. To illustrate, it is less incautious to decide after two fish if the subjective probability is estimated for example at 90% versus if the subjective probability for the same information is estimated at 70%. Here, the decision threshold is more sensitive than the conventional JTC index which may even obscure incautious decision making. Similar results were collected in a recent study [49] that employed a computerized beads task variant with sheep in two different colors as stimuli: schizophrenia patients assigned lower overall probability estimates than healthy subjects, but both groups yielded comparable results on JTC and DTD. This could have been mistaken as a normal decision making in task variants without concurrently collecting probability scores. However, the decision threshold was significantly lower in the patient sample suggesting risky decision making. Importantly and in support of this, the aforementioned study by Andreou et al. [41] found comparable JTC parameters between schizophrenia patients versus controls but a lower decision threshold in patients for the same reasons. 

For future research we would like to encourage researchers interested in JTC to pay a closer look at specific task demands, context effects and patients' attitudes and expectancies. Across-task differences implicitly considered negligible and minor such as mode of presentation (computer, real jars) and test battery (e.g., task administered as a single test versus part of a larger battery that might have cautioned the patient from making hasty decisions) may well have a serious impact on performance. In our view the main question is not whether or not patients show jumping to conclusions but under which conditions this is the case and under which conditions it is not. 

## Figures and Tables

**Table 1:** Demographic, psychopathological, and experimental characteristics of the two samples. Percentages, means, and standard deviations (in brackets).

	Traditional variant (*n* = 961)	Extended variant (*n* = 938)	Statistics
Demographics			
Gender (female/male)	61%/39%	60%/40%	*χ* ^ 2^(1) = 0.74; *P* > .7
Age in years	43.15 (15.72)	43.00 (15.12)	*t*(1897) = 0.24; *P* > .8
Educational level (below 13th grade/13th grade/university degree)	35%/27%/38%	37%/26%/37%	*χ* ^ 2^(1) = 0.29; *P* > .8
Paranoia Checklist			
Total score	26.44 (11.67)	26.88 (11.32)	*t*(1897) = 0.82; *P* > .4
Unspecific suspiciousness (regression score; see factor analysis)	−.02 (1.00)	.02 (.99)	*t*(1897) = 0.81; *P* > .4
Psychotic paranoia (regression score)	−.00 (1.01)	.01 (.99)	*t*(1897) = 0.29; *P* > .7
Probabilistic reasoning			
JTC (1st fish)	37%	66%	*χ* ^ 2^(1) = 163.98; *P* < .001
JTC (1st or 2nd fish)	52%	74%	*χ* ^ 2^(1) = 99.63; *P* < .001
Draws to decision	3.13 (2.56)	2.18 (2.29)	*t*(1897) = 8.49; *P* < .001
Decision threshold in %	—	73.93% (19.56)	—

Note. JTC: jumping to conclusions.

**Table 2:** Comparison between high (≥2 SD) and low scorers (≤0.5 SD) on the Paranoia Checklist with respect to performance on the probabilistic reasoning tasks. Percentages, means, and standard deviations (in brackets).

Traditional variant	High scorers (*n* = 68)	Low scorers (*n* = 485)	Statistics
Draws to decision	2.65 (2.68)	3.46 (2.62)	*t*(551) = 2.37; *P* = .02
JTC (1st fish)	60%	29%	*χ* ^ 2^(1) = 27.18; *P* < .001
JTC (1st or 2nd fish)	63%	45%	*χ* ^ 2^(1) = 7.82; *P* = .005
Initial probability (after the 1st fish)	**—**	**—**	**—**
Decision threshold in %	**—**	**—**	**—**

Extended variant	High scorers (*n* = 68)	Low scorers (*n* = 449)	Statistics

Draws to decision	1.76 (2.07)	2.31 (2.28)	*t*(515) = 2.01; *P* = .047
JTC (1st fish)	81%	61%	*χ* ^ 2^(1) = 10.27; *P* = .001
JTC (1st or 2nd fish)	85%	71%	*χ* ^ 2^(1) = 6.40; *P* = .01
Initial probability (after the 1st fish)	62.95 (24.00)	69.20 (18.63)	*t*(497)* = 2.39; *P* = .05
Decision threshold in %	65.21 (24.80)	75.63 (18.42)	*t*(486)* = 3.94; *P* < .001

Note. SD: standard deviation; JTC: jumping to conclusions; *some subjects did not provide probabilistic estimates.

**Table 3:** Factor solution for the 18 items of the Paranoia Checklist. The first factor is named “unspecific suspiciousness,” the second factor “psychotic paranoia.” Factor loadings above 0.6 are set in bold type.

Paranoia Checklist items	Components	
	Factor 1 unspecific suspiciousness	Factor 2 psychotic paranoia
There might be negative comments being circulated about me.	**.830**	.123
Bad things are being said about me behind my back.	**.813**	.228
People communicate about me in subtle ways.	**.748**	.345
People deliberately try to irritate me.	**.738**	.320
People might be hostile towards me.	**.729**	.306
I need to be on my guard against others.	**.721**	.138
Someone I know has bad intentions towards me.	**.663**	.430
People are trying to make me upset.	**.663**	.353
Strangers and friends look at me critically.	**.653**	.247
People would harm me if given an opportunity.	**.638**	.470
People are laughing at me.	**.610**	.354
I can detect coded messages about me in the press/TV/radio.	.131	**.865**
I am under threat from others.	.222	**.844**
There is a possibility of a conspiracy against me.	.367	**.790**
My actions and thoughts might be controlled by others.	.245	**.780**
Someone I don't know has bad intentions towards me.	.444	**.653**
I have a suspicion that someone has it in for me.	.578	.594
I might be being observed or followed.	.499	.560

**Table 4:** Correlation between different parameters on the probabilistic reasoning tasks with the two factors and the total score of the Paranoia Checklist across the two subsamples and the total pooled sample. Means and standard deviations (in brackets).

	Unspecific suspiciousness	Psychotic paranoia	Paranoia Checklist total score
Traditional variant			
JTC (1st fish)	−.077*	.221***	.062^+^
JTC (1st or 2nd fish)	−.099**	.154***	.006
Draws to decision	.098**	−.136***	.003
Decision threshold in %	—	—	—
Extended variant			
JTC (1st fish)	−.025	.142***	.059^+^
JTC (1st or 2nd fish)	−.035	.132***	.046
Draws to decision	.012	−.122***	−.059^+^
Decision threshold in %	−.014	−.186***	−.119***
Entire sample, both versions pooled			
JTC (1st fish)	−.044^+^	.177***	.064^(∗∗)^
JTC (1st or 2nd fish)	−.063**	.142***	.028
Draws to decision	.054*	−.129***	−.029
Decision threshold in %	—	—	—

Note. JTC: jumping to conclusions.

^+^ *P* ≤ .1, *P ≤ .05, **P ≤ .01, ***P ≤ .001.
